# Simultaneous wastewater treatment and energy harvesting in microbial fuel cells: an update on the biocatalysts

**DOI:** 10.1039/d0ra05234e

**Published:** 2020-07-08

**Authors:** Yajing Guo, Jiao Wang, Shrameeta Shinde, Xin Wang, Yang Li, Yexin Dai, Jun Ren, Pingping Zhang, Xianhua Liu

**Affiliations:** Tianjin Key Lab. of Indoor Air Environmental Quality Control, School of Environmental Science and Engineering, Tianjin University Tianjin 300354 PR China lxh@tju.edu.cn; College of Food Science and Engineering, Tianjin Agricultural University Tianjin 300384 PR China zpp@tjau.edu.cn; Department of Microbiology, Miami University Oxford OH 45056 USA

## Abstract

The development of microbial fuel cell (MFC) makes it possible to generate clean electricity as well as remove pollutants from wastewater. Extensive studies on MFC have focused on structural design and performance optimization, and tremendous advances have been made in these fields. However, there is still a lack of systematic analysis on biocatalysts used in MFCs, especially when it comes to pollutant removal and simultaneous energy recovery. In this review, we aim to provide an update on MFC-based wastewater treatment and energy harvesting research, and analyze various biocatalysts used in MFCs and their underlying mechanisms in pollutant removal as well as energy recovery from wastewater. Lastly, we highlight key future research areas that will further our understanding in improving MFC performance for simultaneous wastewater treatment and sustainable energy harvesting.

## Abstract 2

This review provides an update on MFC-based wastewater treatment and energy harvesting research, and analyze various biocatalysts used in MFCs.

## Graphical abstract

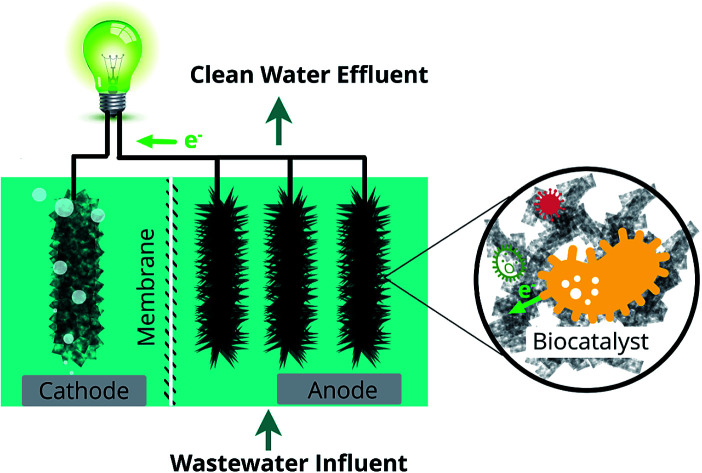

## Introduction

Modern society is burdened by the exhaustion of fossil fuels and environmental pollution, and needs technological inventions to supply renewable energy and clean water. It is well known that a large quantity of untreated or inadequately treated wastewater is discharged into the environment, posing a great risk to the ecological system. Although wastewater contains a large amount of toxic chemicals and biological substances, proper treatment allows its safe usage as fertilizer and potential energy source.^1^ Microbial fuel cells (MFCs) have been reported to treat a wide range of wastewater and are capable of converting the energy contained in wastewater directly into electricity and useful chemicals like H_2_, H_2_O_2_, CH_4_, *etc.*^2,3^ Therefore, wastewater treatment has the potential to become a sustainable process wherein pollutant removal and energy harvest can be achieved simultaneously.^4^

**Fig. 1:**
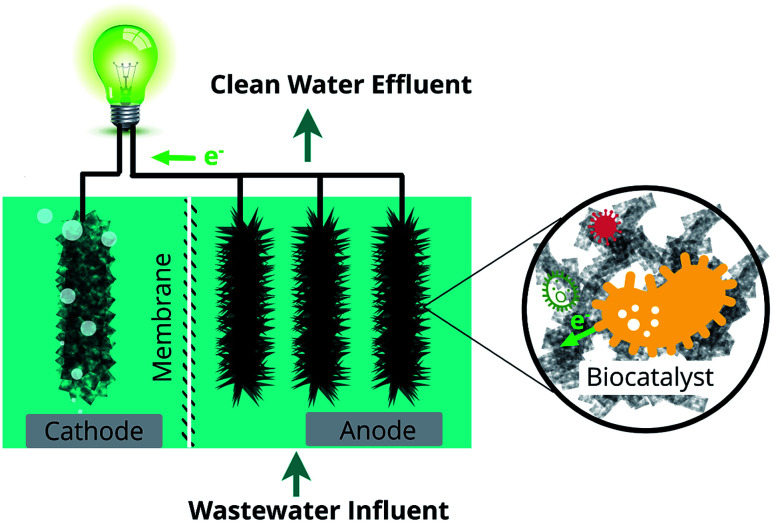
Schematic of using MFC for simultaneous wastewater treatment and energy recovery.

**Fig. 2:**
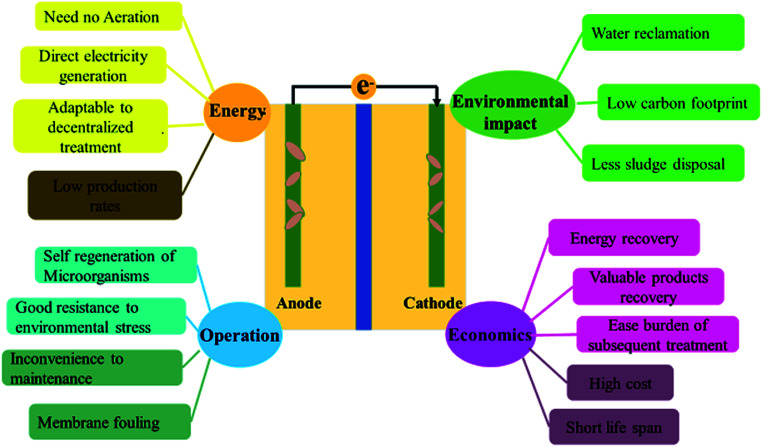
Advantages and disadvantages of MFC technology for treating wastewater.^10^

Microbial fuel cell (MFC) is a bio-electrochemical system that can convert chemical energy to electrical energy through microbial catalysis at an electrode. Pollutants in the wastewater, containing carbon, nitrogen, phosphorus, or heavy metals, can be degraded/stabilized in the chambers of MFC.^5–8^ Simultaneously, the chemical energy trapped in these compounds is converted into electricity (Fig. 1). Among various wastewater treatment techniques, such as chemical treatment, aerobic treatment, anaerobic digestion, and membrane filtration, MFC is considered as a promising technology with the dual purpose of pollutant removal and energy recovery.^9^ MFCs gain a competitive advantage over other water treatment technologies due to their unique features such as huge energy benefits, less environmental impact, good operating stability, and high economic efficiency^10^ (Fig. 2). Compared to aerobic treatment, MFCs produce less sludge and reduce energy consumption.^11^ It is also superior to anaerobic digestion technology owing to its operation flexibility in relatively extreme conditions, like low temperatures (<20 °C) and low substrate concentrations.^12^ However, operation of MFCs also suffers from multiple setbacks such as short life span, high cost, low production rates, limited efficiencies, membrane fouling, instability, and inconvenience in maintaining microbe-based systems (Fig. 2).^10^ The life span of MFCs has always been a concern,^13–15^ which is largely determined by the stability of cathode catalysts and membrane deterioration in most cases.

The relationship between electricity production and wastewater treatment in MFC was first established in 2001 where starch industrial wastewater was used as fuel in MFC for electricity generation.^16^ Since then, MFCs were widely used to remove various pollutants in wastewater. Over the years, researchers have made tremendous progress on structural design and electrode material optimization to enhance MFC performance.^17,18^ However, the systematic information on MFC biocatalysts is still lacking in terms of its importance in simultaneous pollutant removal and energy production. This review aims to fill in this gap and highlight key future research areas to further improve their performance.

## Biocatalyst action mechanisms in MFCs

Many types of biocatalysts have been proven to degrade contaminants and/or generate electricity in MFCs. They have different distributions and roles in different MFC configurations. Interspecific cooperation among pollutant-degrading bacteria, electrogenic bacteria, and other minority bacteria occurs in the MFC systems. First, the pollutant-degrading bacteria on the in the microbial community of an electrode promoted the initial transformation of pollutants. Further, electroactive bacteria and other bacteria degrade the biodegradable ring decomposition products enabling inter-species association. This could explain the enhanced removal efficiency and power generation performance in MFCs.^19^ Each specie plays a specific role in a mixed-culture community, establishing synergy in pollutants degradation, electricity generation, and/or the protection of the community against harmful environmental conditions.^20^

The possible mechanisms of removing pollutants in MFCs can be summarized as follows: when it comes to nitrogen removal,^21–23^ ammonia is oxidized to nitrite and/or nitrate by nitrifying bacteria, such as *Nitrosomonas* sp., *Aridibacter*, *Nitrospira*, and *Bacillus thuringiensis*. Then, nitrite and/or nitrate are deoxidized serially into N_2_ by some denitrifiers, such as N*itratireductor* sp, *Thauera*, *Thiobacillus*, and *Geobacter*; with respect to phosphorus, it can be removed/recovered by chemical precipitation and microbial absorption in MFC. The specific metabolic process in the MFC differs depending on the type of organic pollutants and the MFC operating conditions. MFCs has two positive effects on organics degradation:^24^ First and foremost, the activity and abundance of pollutant-degrading bacteria can be promoted by MFC systems; and secondly, the pollutants in wastewater can be adsorbed and enriched on the surface of the electrodes. Thus, in theory, the problem of low concentration of substrate in the aqueous phase can be partially circumvented, and the removal rate would be accelerated. When it comes to metabolic pathways of organic pollutants in MFC, some researchers believe that the presence of electrodes speeds up microbial degradation, and bacteria use the same metabolic pathways in the absence and presence of electrodes.^25^ However, other researchers propose that the degradation pathway of organic pollutants may be changed in MFCs.

**Fig. 3:**
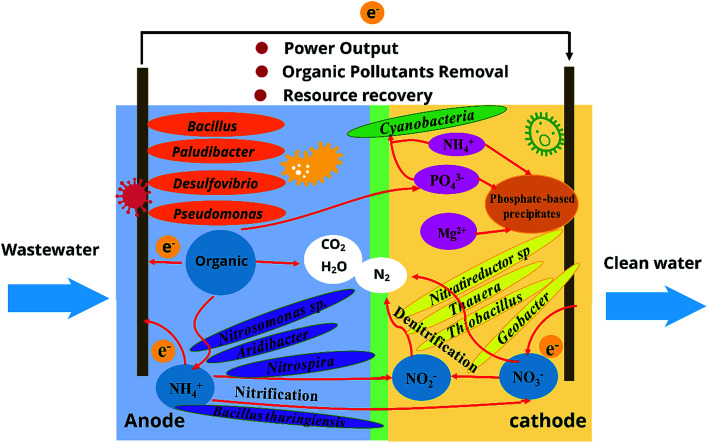
Proposed mechanisms for simultaneous pollutants removal and energy recovery by using MFC.

A schematic illustration on simultaneous pollutant removal and energy generation in terms of key functional biocatalysts and combined interactions in MFC chambers is proposed in Fig. 3.^21–24,26^ The cooperation between pollutant-degrading members and electrogenic members is crucial for achieving pollutant removal and energy production concurrently through MFCs. Pollutants removal process in MFCs can be illustrated as follows:

1 Anode: organic pollutants → CO_2_ + H^+^ + e^−^

2 Anode: NH_4_^+^ + HO^−^ → NO_2_^−^/NO_3_^−^ + H^+^ + e^−^

3 Cathode: NO_2_^−^/NO_3_^−^ + H^+^ + e^−^ → N_2_↑ + H_2_O

4 Cathode: H_2_O + O_2_ + e^−^ → OH^−^

5 Cathode: Mg^2+^ + NH_4_^+^ + PO_4_^3−^ + OH^−^ → MgNH_4_PO_4_·6H_2_O↓

6 Cathode: O_2_ + H^+^ + e^−^ → H_2_O

7 Cathode: NH_4_^+^ + PO_4_^3−^ + CO_2_ + H_2_O → microbial biomass

In addition, electrode material, substrate, temperature, pH, and various other factors, which will be discussed in detail below, can impact microbial activity and their mechanisms. Most of the organic matter in wastewater is unstable and readily decomposed by MFC biocatalysts. It is a source of nutrients for many microorganisms and is readily converted to simple organic acids *via* fermentation, such as acetate. These organic acids and their metabolic intermediates (H_2_, formic acid, *etc.*) produced from fermentation could be further consumed by electro-active microorganisms for nitrogen removal and electricity generation. The complexity of substrate fermentation and the variety of metabolites and intermediates could shape different microbial communities and alter their functions in MFC. There may exist various types of interactions among biological processes, such as uptake of C, N and P nutrients, biomass synthesis and degradation, nitrification, denitrification, bio-mineralization, and energy production. These interactions play significant roles in efficient pollutant removal and energy recovery.

## Research status of MFC for simultaneous wastewater treatment and energy recovery

**Fig. 4:**
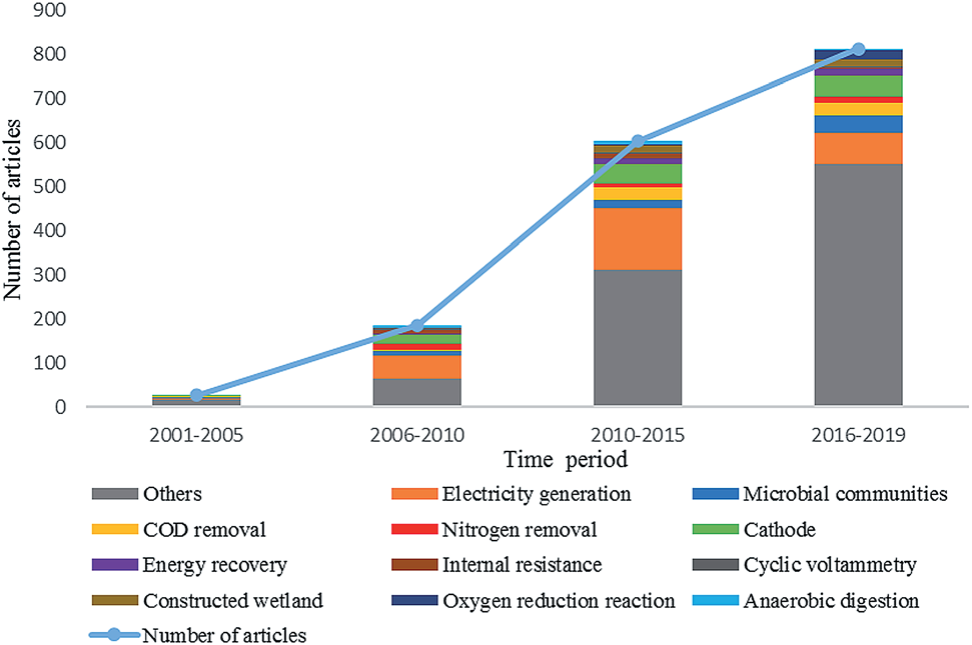
Research tendencies of wastewater treatment by MFC from 2001 to 2019.

**Fig. 5:**
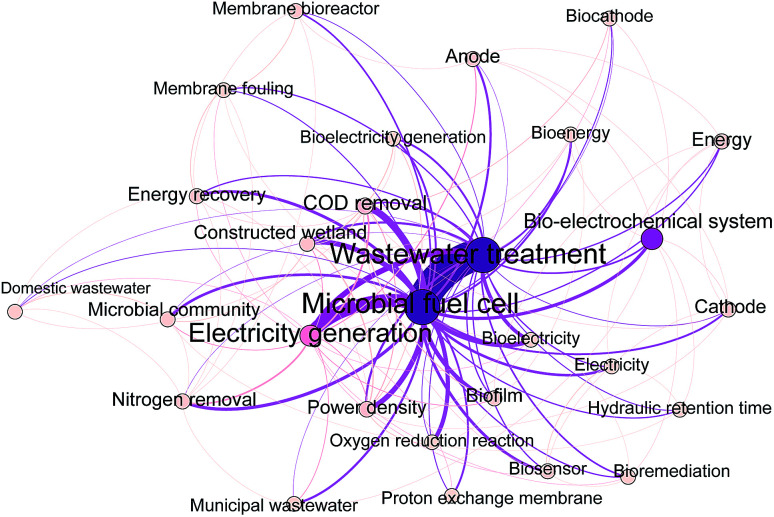
Co-occurrence network analysis of keywords in publications on wastewater treatment by MFC from 2017 to 2019. Each keyword on the map is displayed as a node, with size determined by the occurrence. Keyword relationships are shown as edges of varying thickness determined by the co-occurrence.

To identify research themes and tendencies of MFC studies for wastewater treatment and energy recovery, we conducted a bibliometric analysis. A total of 1626 literatures were retrieved from the web of science database with the keywords of “microbial fuel cell” and “wastewater treatment”. These data were further analyzed by using the software Bibexcel. Fig. 4 shows the annual article numbers on MFC and wastewater treatment from 2001 to 2019. The article numbers and research themes are rising gradually, however, the top ten themes have been relatively stable. These themes focus on electricity harvest (electricity generation, and energy recovery), pollutants removal (COD removal, and nitrogen removal), biocatalyst (microbial communities), MFC configuration and performance characterization (cathode, oxygen reduction reaction, internal resistance, and cyclic voltammetry), and combination of MFC with traditional techniques (constructed wetland, and anaerobic digestion). To emphasize the emerging directions in these fields, a co-occurrence network analysis of keywords from 2017 to 2019 was further conducted (Fig. 5). These data indicate that MFC has great potential for wastewater treatment and sustainable energy harvest, and the tendency of MFC research shows features such as comprehensive technology, practice-orientation, and diversification.

Nevertheless, the present studies on the biocatalysts in MFC are still insufficient. At present, researches on biocatalysts in MFC were mostly limited to biofilm analysis. Although there were some reviews on the varieties of microbial species in MFCs,^27^ detailed information on their roles in removing target pollutants and the underlying mechanisms is still elusive. To overcome this shortcoming, this review describes the role of MFC's microbiome in the degradation of organic matter, nitrogen compounds, and removal of phosphorous along with the action mechanisms.

## Application of biocatalysts in MFCs

Biocatalysts are the basis of MFCs and have a huge impact on their performance. Different types of biocatalysts have different electron transfer mechanisms and pollutant degradation capabilities, which directly affect the MFC's performance in electricity production and pollutant removal.^28^ Therefore, it is imperative to screen and identify microbes that can efficiently degrade pollutants and generate electricity, and explore the possible mechanisms of cooperation between different microorganisms are important for MFC development.

### Biocatalyst in MFCs

MFCs can be classified according to the number of chambers into single-chamber MFCs (SCMFCs) and dual-chamber MFCs (DCMFCs). Both have two electrodes: an anode and a cathode.^29^ Two-chamber MFCs normally have a proton exchange membrane (PEM) physically separating anode and cathode chambers, but allows protons to travel through towards the cathode.

In the MFC system, electrochemically active microorganisms (EAMs) act as biocatalysts, which transfer electrons obtained during degradation of pollutants to an extracellular electron acceptor thereby simultaneously producing energy.^30^ In wastewater fed MFCs, mixed microbial cultures are often used. EAMs for wastewater treatment can be enriched either from activated sludge or various natural environments, such as soil, sediments, and water bodies. For example, Vijay *et al.* in 2019 developed a MFC containing denitrifying microbial consortia from cow manure and soil, which was used to remove nitrate and nitrogen successfully.^31^ The frequently used microorganisms in the MFCs belong to *Shewanella*, *Proteobactor*, and *Pseudomonas* genus.^32^ Digested sludge and anaerobic compost^33,34^ contains a large amount of EAMs and can be used as a screening source for biocatalysts.

Biocatalyst is one of the most important factors affecting overall MFC performance.^35^ Some microorganisms in MFC can increase power generation, while some microorganisms play a fundamental role in removing pollutants from wastewater.^36^ In 2017, Michael J. McAnulty *et al.* created an MFC with a synthetic consortium consisting of an engineered archaeal strain and *Geobacter sulfurreducens*, which allows direct conversion of methane into electric current.^37^ Marassi *et al.* in 2019 employed a consortium of fermenting and metal-reducing bacteria in MFC to treat dairy wastewater.^38^ These reports demonstrate that changing the composition of biocatalysts is a very viable method to target different contaminants while simultaneously generating electricity.

### Factors affecting biocatalyst activity

Various factors influence biocatalysts activity in MFCs including electrode material, substrate, pH, temperature, inoculum source, and nature of pre-enrichment of inoculum. Among them, electrode material and substrate are the most controllable factors.

### Electrode material

The electrode material plays a significant role in enhancing and maintaining biocatalytic activity.^39^ Graphite, graphite felt, carbon paper, carbon cloth, platinum (Pt), Pt black, reticulated vitreous carbon are commonly used materials for an electrode. To enhance the transfer of electrons from biocatalysts to the electrode, the surface of an electrode can be modified to become favourable habitats for biofilms. For instance, Li *et al.* in 2019^40^ used molybdenum dioxide (MoO_2_) nanoparticles dispersed carbon nano-rods as anode material. Due to its excellent biocompatibility, MoO_2_ anode can enrich electroactive bacteria. Moreover, electrode surface modification can enhance reaction kinetics. Coating bacteria with metal nanoparticles promote the transfer rate of an electron from biocatalysts to an electrode.^41^ Metallic nanoparticles can work as connectors between enzyme active sites and electrodes, thereby enhancing the electron turnover rate and achieving efficient electron transfer from microbes to the electrode.^42^ Additionally, the influence of electrode materials on biocatalyst activity can be reflected in power density. It was reported that modification of electrode with graphene oxide can significantly improve MFC power density.^43^

### Substrate

The substrate is another vital factor impacting biocatalyst activity and the proper functioning of the MFC system.^44,45^ The type of substrates can affect the biological properties and the enrichment of bacterial community structure of biocatalysts in MFC.^46^ The different substrates may trigger a specific microbial metabolism mechanism which also affects the metabolism of organic and electronic transfer process consequently.^47^ For example, Sotres *et al.* in 2019^48^ showed changes in the microbial community structure of biocatalyst and MFC performance when the MFCs fed was replaced with synthetic wastewater or pig slurry as substrate.^30,35,49^ An earlier report by Tian *et al.* in 2017,^50^ used varied concentrations of potato pulp wastewater as substrate of MFC to generate electricity. Results indicated that the substrate concentration greatly affected the power output of MFCs, and the predominant populations of biocatalyst distinct significantly from each other under different substrate concentrations.

Co-metabolism, which can be realized by co-substrates, is regarded as one of the feasible ways to improve degradation of recalcitrant pollutants in MFC.^51^ The positive effect of co-substrates could be attributed to the oxidizing enzyme induction and bacteria proliferation supported by the biodegradable carbon resource,^52^ and the detailed mechanism of how co-substrates promote the degradation in MFC need further study. Buitrón *et al.* discovered that the use of acetate as co-substrate improved simultaneous electricity generation and phenol degradation by *Pseudomonas*, *Geobacter* and *Shewanella* in MFC.^53^ Shen *et al.* found that with acetate as co-substrate, not only the electricity production capacity and the electron transfer efficiency in MFCS was enhanced, but also phenol degradation was promoted.^19^ Besides, it was reported that using biodegradable organics as co-substrate could enhance bacterial metabolism and accelerate 2,4,6-trichlorophenol degradation.^46,54^ The co-substrate also show the positive effect on the degradation of *p*-nitrophenol by the anode functional bacteria of the genera *Corynebacterium*, *Comamonas*, *Chryseobacterium* and *Rhodococcus*.^55^

### Other factors

Besides the electrode material and substrate, additional factors influence MFC performance for wastewater treatment and energy production. In an MFC, pH maintains the equilibrium in the redox conditions and is a crucial factor affecting biofilm formation.^56^ Patil *et al.* (2011)^57^ and Margaria *et al.* (2017)^58^ have studied the influence of pH on MFC, showing that pH is important for the microbial community structure and performance of electroactive biofilms. The pH values can influence both the optimal growth of microorganisms and the metabolic activity of substrates, consequently effecting the electron and proton generation mechanisms. Generally, alkaline and neutralized condition is preferable for improving MFC performances. For example, Marashi *et al.*^59^ used purified terephthalic acid wastewater as a fuel in a MFC, and found the MFC performed best at pH 8.5 than at pH 7.0 and pH 5.4. Margari *et al.*^58^ investigated the effect of pH on MFCs inoculated with marine consortia. They found that as soon as the pH deviated from neutrality it affected MFCs' performances. Alkaline conditions with pH values between 8 and 10 corresponded to the formation of a denser biofilm gave the best performance in terms of maximum power density. Temperature is another important factor for biofilms formation and electro-catalytic performance. Patil *et al.* in 2010^60^ found that the MFC used in the study achieved the maximum power density at 35 °C between 5 °C and 45 °C. Inoculum source, nature of pre-enrichment, and pure/mixed microbial culture are also important factors contributing to the MFC performance.^61–63^ For example, the microbial community structure of biocatalyst differed largely when anaerobic sludge and digester sludge was used as the inoculum in MFC.^30^

## Biocatalysts for organic carbon pollutants removal in MFCs

MFC has become a promising solution for wastewater treatment and is regarded as an eco-friendly and sustainable method.^24^ Microorganisms in MFCs have dual roles: degrading pollutants and producing electricity. The COD removal rate is typically used to evaluate the removal of organic matter from wastewater. Various organic matter can be used as a substrate by MFCs, such as carbohydrates and hydrocarbons, which are discharged from domestic activities and numerous industrial sectors such as the food processing industry, dye chemical industry and petrochemical industry.^35,64–66^

Most of the organic matter in wastewater can be nutrient for many fermentative microorganisms and is easily converted to simple organic acids, such as acetate. In the anodic chamber of MFC, some facultative and obligate anaerobic bacteria can perform fermentation, in which the protons and electrons removed during the oxidation of the organic pollutants are transferred to their metabolic intermediates. The fermented products and associated metabolites are further consumed by electroactive microorganisms. Many electroactive microorganisms can perform anaerobic respiration while generating electricity, converting organics pollutants in wastewater to CO_2_. However, some industrial wastewaters may contain refractory organics that resist biological degradation or toxic components that interfere with the activity of biocatalysts.^20^

Researchers have done large amount of work to unveil microbial community structures and search for microorganisms with a high capacity in removing contaminants and generating electricity. Different biocatalysts play different roles in the process of wastewater treatment. Microbial community structure can be altered depending on the types and concentrations of pollutants.^67^ Most of the studies focused on generating more power by efficiently decomposing organic matter in wastewater, however, only one kind of organic pollutant was studied at a given time. The COD removal rate in MFC is influenced by many factors, such as microbial communities, electrodes, and substrate concentrations.^68^ Furthermore, it is difficult to achieve the maximum power output and the highest COD removal rate simultaneously.^69^

Table 1 list recent studies in using MFC to remove organic pollutants from wastewater. These studies indicated that besides SCMFCs and two-chambered MFCs (TCMFCs/DCMFCs), integrated MFC systems also have the potential to be used in wastewater treatment. The integrated systems include sediment microbial fuel cells (SMFCs), MFCs coupled to constructed wetlands (CW-MFCs), desalination MFCs (DS-MFCs), membrane bioreactor MFCs (MBR-MFCs), algae-MFCs (AMFCs) and photo-MFC (P-MFC), expanding the use of MFCs in wastewater treatment. In addition, both pure and mixed cultures can be used in MFCs to remove organic pollutants (Table 1). MFCs with mixed-culture biofilm communities perform better in comparison to pure-culture biofilms, however, pure cultures are better to elucidate the mechanism, chemical interactions, and bacterial growth characteristics. A reasonable explanation for this observation is that MFCs with mixed-culture biofilms have both high tolerance and metabolism of complex substrates due to the metabolic cooperation among different microorganisms.^77,78^

**Table 1:** Studies regarding organic carbon pollutants removal in MFC^a^

MFC type	Organic pollutant	Source of inoculation	Substrates	Microorganisms	Electrode	Performance		Ref.
						Removal efficiency	*P*_max_	
SCMFC	COD	Anaerobic sludges	Effluent from the primary sedimentation tank	Not mentioned	Anode: air-cathode, cathode: ammonia-treated graphite fiber brush	COD removal: 25.8%	422 mW m^−2^	70
Tubular DCMFC	2,4,6-Trichlorophenol	Anaerobic digester	Wastewaters	*Delftia* sp., *Comamonas* sp., *Variovorax paradoxus*, *Brevundimonas diminuta*, *Azoarcus* sp., *Desulfovibrio intestinalis*, *Cytophaga* sp.	Anode: graphite fiber, cathode: graphite felt	0.10 mol m^−3^ d^−1^	2.6 W m^−3^	71
DCMFC	Dichlorophenol	*B. subtilis*	Dichlorophenol	*Bacillus subtilis*	Anode and cathode: carbon cloth	Over 60%	9.5 mW m^−2^	72
DCMFC	COD	Anaerobic sludge	Glucose	Not mentioned	Proton exchange membranes, electrode: graphite plates	72%	60 mW m^−2^	73
SCMFC	COD	Municipal sludge	Synthetic wastewater	Not mentioned	Anode: carbon felt and cathode: carbon clothe, graphene oxide hybridized MgO	COD removal: 79.5%	755.63 mW m^−2^	74
P-MFC	COD	*Spirulina*	Swine-farming wastewater	Cathode: *Spirulina*	Anode: carbon cloth, cathode: activated carbon/PTFE mixture	COD removal: 89%	850 mW m^−2^	75
SCMFC	COD and phosphorus	Anaerobic sludge	Synthetic wastewater	*Thauera*, *Trichococcus*, Rhodocyclaceae	Anode: carbon felt, cathode: air-cathode	More than 97%	4.38 W m^−3^	67
DCMFC	2,4-Dichlorophenol	Domestic wastewater	Synthetic wastewater	*Arcobacter*, *Aeromonas*, *Pseudomonas*, *Acinetobacter*, *Cloacibacterium*, and *Shewanella* sp.	Anode: carbon cloth, cathode: platinised titanium (Pt/Ti) plate	62%	66 mW m^−2^	76
Photo-SCMFC	2,4,6-Trichlorophenol	Municipal wastewater	Municipal wastewater	*Geobacter*, *Pseudomonas*, *Rhodococcus*	Anode: carbon felt with photocatalyst, cathode: air-cathode	79.3% of TCP removal	19.8 W m^−3^	54

a SCMFC: single-chamber microbial fuel cells; DCMFCs: dual-chamber microbial fuel cells; P-MFC: photoautotrophic microbial fuel cells; photo-MFC: photocatalytic microbial fuel cells; COD: chemical oxygen demand.

In general, the microbial community diversity in MFCs treating wastewater can be functionally categorized into pollutant degraders, electrogenic bacteria, and the minority of related bacteria. The complex syntrophic synergy among MFCs microbial communities significantly enhances the degradation of organic carbon pollutants and power generation performance of the MFCs systems.^55^ For instance, previous studies indicated that the synergistic interactions between fermentative and electrogenic bacteria can improve the degradation of contaminants.^55,79,80^ Moreover, non-electrogenic microbes are essential for the microbial ecology of MFCs. They provide a local anaerobic environment in mixed culture systems facilitating higher power production using anaerobic electroactive bacteria when compared to pure cultures grown in aerobic conditions.^20,81,82^ Also, Table 1 shows multiple studies that use anaerobic sludge as a source of inoculation for treating organic pollutants wastewater with MFCs. All studies reviewed in Table 1 signifies a trend wherein bioelectrodes under anaerobic conditions exhibited higher degradation rates, whereas aerobic conditions achieved higher maximum powers.^71^ Henceforth, microbes known with the ability to degrade specific pollutants, used in MFCs, will be discussed in detail.

Polycyclic aromatic hydrocarbons (PAHs) are ubiquitous organic pollutants in industrial wastewater,^83^ difficult to degrade and dangerous to plants, animals, and humans.^84^ In 2010, Zhang *et al.* found that aromatic hydrocarbon, toluene, can be degraded into CO_2_ by MFCs using *Geobacter metallireducens* as biocatalyst.^85^ Yun *et al.* in 2017 reported that the use of microbes from phylum Proteobacteria, Bacteroidetes, and Firmicutes in biocathodes significantly accelerated the reduction of the nitroaromatic compound like nitrobenzene (NB).^86^ Moreover, species belonging to genus *Bacillus*, *Paludibacter*, *Desulfovibrio*, and *Lactococcus* have been proven to be PAH degraders in MFCs.^87–89^ Researchers found that the use of *Enterobacter cancerogenus* BYm30 in MFC results in the degradation of phenols.^90^ And *Pseudomonas* and *Geobacter* sp. often dominant in MFCs fed by acetate and can also degrade phenolic contaminants.^54,91^ Although *Bacillus subtilis* is inefficient in generating electricity,^92^ it plays a role in scavenging 2,4-dichlorophenol (2,4-DCP).^93^*Arcobacter*, *Aeromonas*, *Acinetobacter*, *Cloacibacterium*, and *Shewanella* were also reported to be dominant bacteria for 2,4-DCP degradation in MFCs.^76^

Polychlorinated biphenyls (PCBs), another class of refractory organic compounds, are widely dispersed in the global ecosystem. It is reported that *Longilinea* spp. can provide electron donors (H_2_) for the dichlorination of PCBs.^94,95^*Clostridium*, *Longilinea*, and *Acetoanaerobium* bacteria can remove PCBs effectively in MFC.^96^ Another study also attributed the enhanced degradation of PCBs in MFC to *Alcanivorax*, *Mycobacterium*, *Parvibaculum*, *Dehalogenimonas*, *Comamonas*, *Hydrogenophaga*, and *Sedimentibacter*.^97^*Actinobacteria*, widely distributed in a PCB-contaminated soil ecosystem,^98^ were detected in MFCs treating PCBs.^97^ Other biocatalysts such as *Gordonia*^99^ and Chloroflexi^97^ can respire with PCBs and thus used to remove PCBs in wastewater.

Beside PAHs and PCBs, biocatalysts in MFC were reported to remove various other refractory organic pollutants. The co-enriched *Paludibacter*, *Desulfovibrio*, and *Lactococcus* were able to degrade aromatic compounds.^100,101^*Thauera* was found to be the dominant genus during the removal of aromatic compounds by MFC.^102^*Rhodococcus* also contribute to the degradation of aromatic compounds in MFCs, specifically chlorophenols.^74,103^ Song *et al.*^104^ demonstrated that *p*-chloronitrobenzene (*p*-CNB) can be removed by MFC using *Pseudomonas fluorescens*.

## Biocatalysts for nitrogen removal in MFCs

There are various forms of MFC available to reduce nitrogen from wastewater: cathodic denitrification-MFC (CD-MFC),^105,106^ anodic denitrification-MFC (AD-MFC),^107^ nitrification-MFC,^108,109^ simultaneous nitrification and denitrification-MFC (SND-MFC), and anammox-MFC.

In the denitrification-MFC, an electron donor (such as carbon substrate) is required to convert nitrate to nitrogen gas, whereas an electron acceptor (such as oxygen gas) is needed to convert of ammonium to the nitrite and nitrate in the nitrification MFC.^110^ Ammonia can also be used as a fuel in nitrification-MFC.^109,111^ He *et al.*^109^ demonstrated the use of ammonia as anode fuel in a nitrification-MFC, and achieved 49.2% and 69.7% removal rates of ammonia nitrogen. CD-MFC is the earliest denitrification-MFC. Both nitrate and nitrite can serve as cathode electron acceptors to generate electricity ^105 106^. For example, Virdis^105^*et al.* designed a CD-MFC to study nitrogen removal efficiency and electricity generation using a biocathode. Nitrate can also be reduced at the anode of MFC. A study conducted by Zhang *et al.*^107^ where the use of AD-MFC accomplished the denitrification rate of 1.26 kg (m^3^ d)^−1^.

Research on SND-MFC has attracted attention in recent years.^112^ The concurrent nitrification and denitrification in an SND-MFC may be due to the stratification phenomenon found in the biofilm growing on cathode.^113^ The outer layer of the biocatalyst comprises nitrifying bacteria that oxidize NH_4_^−^ to NO_3_^−^ aerobically and the inner layer encompasses denitrifying bacteria that convert NO_3_^−^ and NO_2_^−^ to N_2_ in an oxygen-limited environment. Albeit the advantage of SND-MFC, studies also reported its poor removal efficiency.^114–116^ A reasonable explanation for this observation could be the difficulty in maintaining hypoxic and aerobic conditions for the microbes in a single chamber.

Anaerobic ammonium oxidation (ANAMMOX) is an important microbial process in the nitrogen cycle, which converts nitrite (electron acceptor) and ammonium (electron donor) to nitrogen gas.^117^ Anammox-MFC is designed for complete simultaneous removal of ammonia (NH_4_^+^) and nitrite nitrogen simultaneous removal of ammonia (NH_4_^+^) and nitrite nitrogen (NO_2_^−^). Nevertheless, there are relatively few studies on anammox-MFC, and the specific working mechanism is not clear. Further studies should emphasize the identification of dominant bacteria for the electro-anammox process and the underlying mechanisms under anaerobic conditions.^118^

Biocatalysts for nitrogen removal in MFCs can be divided into nitrifying bacteria and denitrifying bacteria.^119^ Both denitrifying and nitrifying bacteria are Gram-negative bacteria and are rich in cytochrome C. These characteristics are analogous to the electrogenic microorganisms implying that denitrifying and nitrifying bacteria have the potential to produce electricity. Most studies have focused on optimizing reactor configuration, electrode construction, and utilizing electron-donating mediators to improve the nitrogen removal efficiency in MFC.^112,124,125^ Key information about biocatalysts associated with nitrogen removal in MFC is still lacking.

Some key nitrifying bacteria and denitrifying bacteria involved in nitrogen removal in MFC can be summarized as follows. Proteobacteria, Chloroflexi, Bacteroidetes, Nitrospirae, and Planctomycetes were found to be the principal bacteria that contributed to nitrogen removal in MFC.^116,126,127^ Gregory *et al.*^128^ proved that the genus *Geobacter* sp. is capable of denitrification by using electrode electrons to reduce nitrate to nitrite. Proteobacteria, Bacteroidetes, and Firmicutes were dominant in MFC, participating in the denitrification process.^129^ Additionally, Gammaproteobacteria and Bacteroidetes capable of autotrophic denitrification participated in nitrogen removal in MFC.^31,130,131^ The genus *Thiobacillus* played a pivotal role in pyrite-driven autotrophic denitrification in MFC.^131^*Pseudomonas stutzeri*, *Exiguobacterium* sp., *Nitratireductor* sp., and *Acidovorax* sp. proved to be the key electroactive denitrifiers in the MFC.^22,31,132–135^ On comparison of the microbial community structure in ordinary MFC without denitrification function and denitrification-MFC, the increase in growth of *Thauera* and *Emticicia* and *Rheinheimera* was reported in the latter.^136^ Li *et al.*^137^ found that *Paracoccus* spp., the main functional bacteria associated with denitrification,^138,139^ were well-enriched in the MFC system. Studies found that denitrification bacteria, including *Zoogloea*, *Rhodobacter*, *Mesorhizobium*, *Hydrogenophaga*, *Brevundimonas*, *Flavobacterium*, *Bosea* and *Bdellovibrio*, potentially cooperate to fulfil NO_2_^−^ and/or NO_3_^−^ reduction process in MFC.^140,141^*Pirellula*, *Nitrospira* and *Nitrosomonas* were found acting as nitrifiers in MFC.^21–23,26,116,119,122,142–144^ Treesubsuntorn *et al.*^145^ found that *Bacillus thuringiensis*, an effective nitrifying bacterium, enhanced both the nitrogen removal efficiency and power density of MFC. *Nitrospira* and *Aridibacter*, known nitrifiers under the aerobic environment,^146^ were the dominant genera in MFC.^116^

Table 2 lists studies on nitrogen removal by using MFC. It can be seen that using *Thauera* as a biocatalyst for wastewater treatment in MFC is a research hotspot recently.^21,22,120,122^*Thauera* is widely found in the denitrification process of wastewater treatment.^147^ Two other studies further proved that *Thauera* contributed to nitrogen removal under strictly anaerobic conditions in MFC.^148,149^ Besides, Table 2 demonstrates that mixed cultures are preferred biocatalysts to be inoculated when using MFC to treat wastewater, although the use of pure cultures is advantageous to delineate the underlying mechanism.^150^ Contrary to organics pollutants, aerobic sludge was preferred when removing nitrogen in MFCs.

**Table 2:** Varied research on nitrogen and phosphate removal by using MFC^a^

MFC type	Pollutant	Source of inoculation	Substrates	Microorganisms	Electrode	Performance		Ref.
						Removal efficiency	*P*_max_	
FA-MFC	Organic and nitrogen compounds	Activated sludge	Domestic wastewater	*Nitrosomonas marina*, *Nitrosomonas* sp. Nm59, *Nitratireductor* sp., *Acidovorax* sp.	Anode: 30% wet-proof carbon cloth with a platinum (Pt) catalyst, cathode: air cathode	COD: 85%, TN: 94%	6.3 W m^−3^	22
DCMFC	NO_3_^−^ and ClO_4_^−^	Activated sludge and excess sludge mixed in 1 : 1 vol ratio	NO_3_^−^ and ClO_4_^−^ mixed in 1 : 1 molar ratio	β-Proteobacteria, *Thauera* and *Thiobacillus*	Anode and cathode: carbon felts	ClO^4−^: 40.97%, NO^3−^: 86.03%	2.2 W m^−3^	120
SCMFC	Nitrogen compounds	Aerobic nitrifying sludge	Synthetic ammonia-contaminated wastewater	*Nitrosomonas*, *Alishewanella*, *Arcobacter*, *Thauera* and *Rheinheimera*	Anode and cathode: active-carbon felt	Ammonia 99.34%, total nitrogen (TN) 99.34%, COD 90.79%	104 mW m^−3^	23
SCMFC	NH_4_^+^-N	Aerobic denitrifying sludge	Synthetic wastewater	Anode: *Thauera*, cathode: *Thauera*, *Nitrosomonas*, *Desulfomicrobium* and *Thiobacillus* (3–5%)	Anode: carbon felt and cathode: air cathode, MnO_2_-catalyst	COD: 90%, ammonia: 98%; TN: 95%	1270 mW m^−2^	21
DCMFC	NH_4_^+^-N and PO_4_^3−^-P	Anaerobic sludge	Municipal wastewater	Not mentioned	Anode: graphite felt, cathode: carbon-fiber brush coated with a titanium bar	NH_4_^+^-N: >97.58%, PO_4_^3−^-P: >94.9% P	Not mentioned	121
DCMFC	Nitrogen, phosphorus and COD	Anaerobic active sludge	Mustard tuber wastewater	*Nitrosomonas* SM1A02, *Thauera*, *Stenotrophomonas*, *Flavobacterium*, *Marinobacter*, and *Thioalkalispira*	Anode and cathode: carbon cloth	Total phosphorus (TP): 80.8 ± 1.0%, COD: >90	Not mentioned	122
DCMFC	Nitrogenous compounds	*Chlorella vulgaris*	Swine wastewater	*Chlorella vulgaris*	Anode: carbon felt, cathode: carbon fiber cloth	Ammonia nitrogen: 85.6%, TN: 70.2%	3720 mW m^−3^	123
P-MFC	Nitrogenous compounds and phosphate	*Chlorella vulgaris*	Municipal wastewater	*Chlorella vulgaris*	Anode and cathode: carbon brushes	NH_4_^+^: 95.9%, TN: 95.1%, PO_4_^3−^-P: 82.7%	466.9 mW m^−3^	26
AD-MFC	Nitrate nitrogen	Cow manure and soil	Municipal wastewater	*Thauera* and *Pseudomona*	Anode and cathode: graphite felt	NO_3_^−^: 0.118 kg m^−3^ d^−1^	4.45 W m ^−3^	31
HD-MFC	Nitrate nitrogen	Cow manure and soil	Municipal wastewater	*Klebsiella* and *Alkaliphilus*	Anode and cathode: graphite felt	NO_3_^−^: 2.06 kg m^−3^ d^−1^	3.02 W m^−3^	31

a FA-MFC: flat-panel air-cathode microbial fuel cells; SCMFC: single-chamber microbial fuel cells; DCMFCs: dual-chamber microbial fuel cells; P-MFC: photoautotrophic microbial fuel cells; AD-MFC: autotrophic cathodic denitrification microbial fuel cells; HD-MFC: heterotrophic cathodic denitrification microbial fuel cells; COD: chemical oxygen demand; TN: total nitrogen.

Microalgae are unicellular eukaryotes that can uptake nitrogen from wastewater.^135^ Microalgal biomass is regarded as a promising and substantial substitute for biodiesel production. Using microalgae as biocatalysts can save energy and compensate for the operational cost of the MFC.^130^ Ma *et al.*^151^ used concentrated *Chlorella* biomass along with a consortium of photosynthetic organisms (such as *Azospirillum* and *Rhizobium*) to remove nitrogen. Results illustrated concomitant removal of nitrogen and electric current generation.^151^ Zhang *et al.*^123^ employed *C. vulgaris* as a biocatalyst in MFC to treat swine wastewater. Their results showed that the maximum power density of the MFC was up to 3720 mW m^−3^ at 240 hours with the removal rate of ammonia nitrogen, total nitrogen (TN), and total organic carbon (TOC) to be 85.6%, 70.2%, and 93.9%, respectively. Compared to standalone MFCs, the microalgae assisted MFC possibly generate greater electricity.^152^

In a word, previous studies have represented the promising prospects of using MFCs for removal of nitrogen. To achieve better performance and expand the application, mechanistic studies on nitrogen removal and practical studies on different MFC configurations should be conducted further.

## Biocatalysts for phosphorus removal and recovery in MFCs

Phosphate is an important non-renewable and depleting resource in nature. However, the large amount of phosphate in wastewater treatment plants is an important unexploited source.^153,154^ A great deal of research has been focused on the removal and recovery of phosphate from wastewater. MFC has proven to be a feasible method for this application.^136,155,156^ Phosphate (PO_4_^3−^) can be removed by microbial uptake in MFCs. For example, microalgae have been used to remove phosphorus in leachate by the assimilation of nutrients into biomass.^157,158^ Wang *et al.*^26^ observed that cyanobacteria can uptake phosphate in a photoautotrophic MFC and remove about 64.1–82.9%. *Desulfomicrobium* has also been reported to have the ability to remove phosphorus in MFC.^26^

Another attractive feature of MFC technologies is their capability to recover phosphorus as fertilizer by producing phosphate-based precipitate in MFC chambers *via* struvite (MgNH_4_PO_4_·6H_2_O) precipitation.^159,160^ This method is very suitable for the treatment of wastewater rich in nitrogen and phosphorous, such as swine wastewater and urine.^161^ The integration of MFCs with struvite precipitation has been beneficial for energy generation and phosphorus recovery from wastewater.^162–165^ By using MFC, Zang *et al.*^166^ successfully recovered 94.6% of phosphate, 28.6% of ammonia, achieved 64.9% COD removal, and power output up to 2.6 W m^3^ during treatment of urine waste. Li *et al.*^167^ recovered phosphorus by the combined application of chemical precipitation and microbial absorption in MFC. Fischer *et al.*^168^ used a three-stage single chamber MFC for phosphorus recovery, and achieved a 78% recovery. Lu *et al.*^164^ designed a three-chamber resource recovery MFC to treat urine-containing wastewater. They recovered N, P, S nutrients and salt in liquid form, which will be an appealing technology for sustainable resource recovery from wastewater.

Therefore, phosphorus can be effectively removed by microbial absorption and recovered by chemical precipitation in MFC. Studies suggest that phosphorus could be removed up to 82% by MFC systems, 40% of which could be recovered as struvite.^169^ With the gradual depletion of phosphorus resources, phosphorus recovery from wastewater by using MFC systems show great potential for sustainable phosphorus supply.

## Biocatalysts for energy generation in MFCs

In addition to the removal of contaminants, the biocatalysts inoculated at the anode and/or the cathode play an important role in electricity generation.^170^ Production of electric current in MFC is influenced by electroactive microorganisms that accomplish the process through at least three ways: electron shuttling *via* cell secreting mediators (*e.g.*, phenazine, quinones), membrane-bound redox proteins (*e.g.*, cytochromes), and conductive pili (or nanowires) observed in wired communities of *Geobacter sulfurreducens*, *Shewanella oneidensis*.^90,171^ Proteobacteria, Acidobacteria, Firmicutes, *Geobacter*, *Shewanella*, *Rhodoferax*, *Aeromonas*, *Pseudomonas*, *Clostridium*, *Rhodobacter*, *Enterococcus*, *Dechloromonas*, *Rhodopseudomonas* and *Desulfuromonas* have been reported to generate electricity in MFCs.^172–178^ Wang *et al.* investigated the treatment of biogas plants wastewater using MFCs. They found that *Pseudomonas* spp. can shorten start-up time and enhance electricity generation.^179^ Studies have also found that the MFCs using mixed microbial cultures as biocatalyst have higher bioenergy output than using pure cultures.^82^ The enhanced performance may be due to the synergistic interactions between biocatalysts.^87,173^

In a MFC reactor, protons generated from an anode would be reduced to hydrogen by electrons under a suitable external voltage in the cathode chamber.^180–183^ However, this process is relatively hard to achieve due to the thermodynamic barrier and the complex involved external circuit. Therefore, coupling MFC with other systems, like microbial electrolysis cell,^184,185^ photoelectrochemical (PEC) system,^186^ has been proposed to produce hydrogen. Recently, Moradian *et al.*^187^ isolated and identified a new exoelectrogenic yeast strain (*Cystobasidium slooffiae* strain JSUX1) that can simultaneously produce bio-hydrogen and bio-electricity in a microbial fuel cell (MFC) when xylose is used as the substrate.

Electricity generation of MFC is affected by many factors such as internal resistance of the system, electrode size, electrode spacing, conductivity, pH, and chemical structure of pollutants, *etc.*^188^ Therefore, their electricity generation potential of different biocatalysts cannot be compared with each other unless they have the same system architecture and physicochemical environment. Zhang *et al.* indicated that the biodegradable properties of the organic compounds can influence the electricity generation in the MFC.^137^ Compared to the refractory organics, the easily degradable compounds showed higher CE and lower internal resistances in the MFCs.^137^

In a word, choice of robust biocatalysts is one of the important considerations for microbial electrogenesis. Novel microbes with efficient electronic transportation and enhancing pollutant clean-up capacities are anticipated by using various technologies to modify the metabolism of pure cultures. Pre-genomic, genomic and post-genomic techniques are valuable for identifying and constructing cooperative microbial communities. Furthermore, the microbe-electrode electron transfer might be more facile by developing electrode modification to provide materials with better affinity to exoelectrogens/electrotrophs.

## Conclusion and prospect

Biocatalyst is one of the major factors affecting overall MFC performance in pollutant removal and energy recovery from wastewater. Our data have shown that MFC does have great potential for wastewater treatment and sustainable energy harvesting with research focusing on features such as comprehensive technology, practice-orientation, and diversification. MFC biocatalysts are capable of removing varied pollutants from wastewaters, and diversity in biocatalysts is due to different electron transfer mechanisms and pollutant degradation capabilities. The composition of microbial communities can change depending on the type and content of pollutants. Electrode material, substrate, temperature, pH, and various other factors influences on biocatalyst activity can further impact the performance of MFC. Many forms of MFCs can be used to treat nitrogen-containing wastewater: cathodic denitrification-MFC, anodic denitrification-MFC, nitrification-MFC, simultaneous nitrification and denitrification-MFC, and anammox-MFC. Phosphorus in the wastewater can be removed and recovered by chemical precipitation and microbial absorption in MFC. Numerous biocatalysts can degrade contaminants and generate electricity in MFCs, and there may exist various interactions among biological entities that influence their functions such as uptake of C, N and P nutrients, biomass synthesis and degradation, nitrification, denitrification, bio-mineralization, and energy production. These interactions played significant roles in efficient pollutant removal and energy recovery.

The following issues involved with biocatalysts should be given priority for significant developments of wastewater treatment in MFC technology:

Biocatalysts play a vital role in pollutant removal and energy recovery. Although EAMs are changing with various pollutants, characteristic taxonomic groups seem to cooperate in pollutant degradation processes. These microbial communities have numerous interactions during various wastewater treatment processes. The interaction mechanism of biocatalysts for pollutants removal and simultaneous energy recovery in MFCs is still unclear, which limits the research on improving the efficiency of decontamination and energy harvesting. Interactions of EAMs and genes involved in the processes need to be further investigated. For example, measures to control competition in biofilm and form an effective microbial population to improve the removal efficiency of pollutants by MFC and elucidating the mechanism for initial competition among various microorganisms during the microbial enrichment process.

(2) Although significant progresses on pollutants removal efficiency and energy recovery have been made, studies on nutrient extraction and recovery from wastewater are least explored. MFCs can act as tools for the conversion of various environmental contaminants into resources. It is essential to study alternative wastewater treatment pathways capable of simultaneous resource capture and utilization, which will contribute to negative carbon emissions.

(3) Using MFC to remove contaminants in wastewater is still at the laboratory scale. The key factor that affects the industrial-scale applications of MFC treating wastewater is the availability of potent microbial strains. Identification of proper microbes involves high-throughput screening of potent biocatalysts, constructing microbial communities, and controlling its composition, structure, and functional activity, such as using metagenomics, associated functional studies to infer community structure and biological processes within the MFCs system, designing synthetic consortia or co-culture for MFC applications. Additionally, interactions of electrode materials with the microbial community should be further explored. The development of potent biocatalysts and low-cost efficient electrode materials is important for the construction of MFCs which can be widely applied in wastewater treatment plants. At the same time, with the development of technology, topics on the mathematical models, stabilization, or simplification of processes like automatic control systems are key areas for future researches.

## Conflicts of interest

There are no conflicts to declare.

## Supplementary Material
